# *Mycobacterium tuberculosis* ClpP Proteases Are Co-transcribed but Exhibit Different Substrate Specificities

**DOI:** 10.1371/journal.pone.0060228

**Published:** 2013-04-01

**Authors:** Yoann Personne, Amanda C. Brown, Dorothée L. Schuessler, Tanya Parish

**Affiliations:** Queen Mary University of London, Barts & The London School of Medicine and Dentistry, London E1 2AT, United Kingdom; University of Delhi, India

## Abstract

Caseinolytic (Clp) proteases are widespread energy-dependent proteases; the functional ATP-dependent protease is comprised of multimers of proteolytic and regulatory subunits. *Mycobacterium tuberculosis* has two ClpP proteolytic subunits (ClpP1 and ClpP2), with both being essential for growth *in vitro*. *ClpP1* and *clpP2* are arranged in an apparent operon; we demonstrated that the two genes are co-expressed under normal growth conditions. We identified a single promoter region for the *clpP1P2* operon; no promoter was detected upstream of *clpP2* demonstrating that independent expression of *clpP1* and *clpP2* was highly unlikely. Promoter activity was not induced by heat shock or oxidative stress. We identified a regulatory region upstream of the promoter with a consensus sequence matching the ClgR regulator motif; we determined the limits of the region by mutagenesis and confirmed that positive regulation of the promoter occurs in *M. tuberculosis*. We developed a reporter system to monitor ClpP1 and ClpP2 enzymatic activities based on LacZ incorporating *ssrA*tag sequences. We showed that whilst both ClpP1 and ClpP2 degrade SsrA-tagged LacZ, ClpP2 (but not ClpP1) degrades untagged proteins. Our data suggest that the two proteolytic subunits display different substrate specificities and therefore have different, but overlapping roles in *M. tuberculosis*.

## Introduction

Tuberculosis is a major health problem causing approximately 1.5 million deaths each year, with one-third of the world’s population estimated to be infected [1]. The genome of *Mycobacterium tuberculosis,* the main causative agent for tuberculosis, encodes 142 proteases [2] including FtsH, the proteasome and the Clp proteases.

Caseinolytic proteases (Clp) are highly conserved serine proteases present in a wide range of bacteria as well as in plants and mammals [3]. Clp are two-component proteases comprising of a catalytic subunit (ClpP) and a regulatory subunit (Clp ATPase). The catalytic subunit is composed of two heptameric rings of ClpP stacked on top of each other forming a cavity where protein degradation occurs.

*M. tuberculosis* contains two *clpP* genes, *clpP1* and *clpP2*, both essential for growth [4]–[6]. Furthermore, a knockdown strain of ClpP1P2 showed reduced growth *in vitro* and lack of replication after infection of macrophages [4]. Unusually, *M. tuberculosis* ClpP1 and ClpP2 can interact to form an active proteolytic complex composed of one ring of ClpP1 and one ring of ClpP2 [6], [7]. The ClpP1 structure was resolved and displays a unique N-terminal configuration [8]. In *M. tuberculosis,* ClpP1 and ClpP2 are highly expressed in both aerobic and hypoxic environments [9]. Diamide, a thiol-oxidative agent, and vancomycin may induce *clpP1* and *clpP2* expression in *M. tuberculosis* and *M. smegmatis* respectively [10], [11].

A novel class of compounds, the acyldepsipeptides (ADEPs), are active against *M. tuberculosis* and also have potent activity against various Gram positive bacteria, including multidrug resistant isolates such as methicilin-resistant *Staphylococcus aureus* (MRSA) [5], [12], [13]. Unusually the ADEPs do not act as inhibitors, but induce unregulated proteolysis by ClpP which recognises and degrades unfolded polypeptides and ultimately results in cell death [14].

ClgR is a transcriptional activator of *clpC* and *clpP1P2* gene expression in *Corynebacterium glutamicum* and *Streptomyces lividans* [15], [16]. Rv2745c, the *M. tuberculosis* ClgR homologue, binds upstream of *clpP1P2* and activates transcription in *M. tuberculosis* [*17*] *.* Deletion of ClgR results in a reduced capacity to replicate in macrophages [18]. Moreover ClgR, as well as the two ClpP proteases, are involved in the reaeration response: they have been found to be induced during the transition of *M. tuberculosis* from bacteriostasis to growth [17].

The presence of two ClpP subunits in *M. tuberculosis* is unusual and intriguing. We are interested in finding the regulation mechanisms controlling their expression and demonstrated a common regulation but different specificities for the two ClpP subunits.

## Materials and Methods

### Culture

*M. tuberculosis* H37Rv was cultured in Middlebrook 7H9 liquid medium supplemented with 10% v/v OADC (oleic acid, bovine serum albumin, dextrose, catalase; Becton Dickinson) or AD (5% w/v bovine serum albumin, 2% w/v glucose) and 0.05% w/v Tween 80, or Dubos liquid medium supplemented with 10% v/v Dubos medium albumin (Becton Dickinson) and 0.05% w/v Tween 80 (DTA) or on Middlebrook 7H10 agar supplemented with 10% v/v OADC. For LacZ reporter system experiments *M. tuberculosis* was grown in 7H9 medium supplemented with 10% v/v BSA, 0.1% w/v succinate and 0.05% w/v Tween 80 plus 0.1% w/v acetamide where indicated. Streptomycin was used at 40 µg/mL and hygromycin at 100 µg/mL where required. Hypoxia was generated in 20 mm sealed glass tubes containing 17 mL DTA with stirring at 150 rpm as per the model of Wayne (head space ratio 1∶2) [19]. For reaeration, 3 mL of hypoxic cultures were used to inoculate 100 mL of fresh medium in 450 cm^2^ roller bottles (5∶1 head space ratio) and incubated with rolling at 37°C. Oxidative stress was generated with 10 mM diamide, vancomycin was used at 6 µg/mL, chlorpromazine at 50 µg/mL and menadione at 10 µg/mL.

### Semi-quantitative RT-PCR

RNA was prepared from *M. tuberculosis* standing cultures during late exponential phase [20] and DNAase treated. cDNA was synthesized from 1 µg of total RNA using SuperScript II RT (Invitrogen) and random primers. PCR was carried out using undiluted cDNA and serial 4-fold dilutions (1∶4, 1∶16, 1∶64, 1∶256, 1∶1024). Cycling conditions were 30 cycles of 94°C for 30s, 56°C for 30s, and 72°C for 1 min using GoTaq PCR Mastermix (Promega). *SigA* was used as an internal RNA control.

### Construction of Plasmids and Recombinant Strains

Two regions upstream of *clpP1P2* were PCR amplified (using reverse primer AGT ACT GCT CAC AGT GGG GCA CCT with forward primer AGT ACT TGA CCG TAT GAC GCT GTA to create P_125_ or forward primer AGTA CTC AGG GCC GCA GTG GAG GC to create P_278_) and cloned into the integrating promoter reporter vector pSM128 [21]. Site directed mutagenesis (SDM) was carried out as described [22].

Protein tags (AADENYA-ASV and AADENYA-LAA) were added to the C-terminal end of *lacZ* in pSM128 by two rounds of PCR using forward primer GGT CTG GTG TCA AAA AGC AGC AAA CGA CGA AAA CTA CGC TTT AGC AGC TTA ATA ATA AC and its reverse complement to add the AADENYA core amino acid sequence, and using primers GGT CTG GTG TCA AAA AGC ATC AGT TTA ATA ATA ACC GGG for the ASV end and GGT CTG GTG TCA AAA ATT AGC AGC TTA ATA ATA ACC GGG for the LAA end and their respective reverse complements. AADENYA-ASV was mutated to AADENYA-GGG by site directed mutatgenesis. The acetamidase promoter (P_ami_) [23] was excised from pFLAME-3-ace [24] and cloned into the *ScaI* sites of the plasmids upstream of *lacZ*.

Over-expressing ClpP1 and ClpP2 strains were obtained by electroporation of the recombinant plasmids pOPPY1, pOPPY2 and pOPPY7 [5] into *M. tuberculosis* and selection with hygromycin.

### Promoter Activity Assays

Sequence-verified plasmids were electroporated into *M. tuberculosis* and transformants were selected on streptomycin. Three independent transformants for each strain were grown as standing cultures; cell-free extracts and β-galactosidase assays were performed as described previously [25].

## Results

### *clpP1* and *clpP2* are Co-transcribed in *M. tuberculosis*

**Figure 1:**
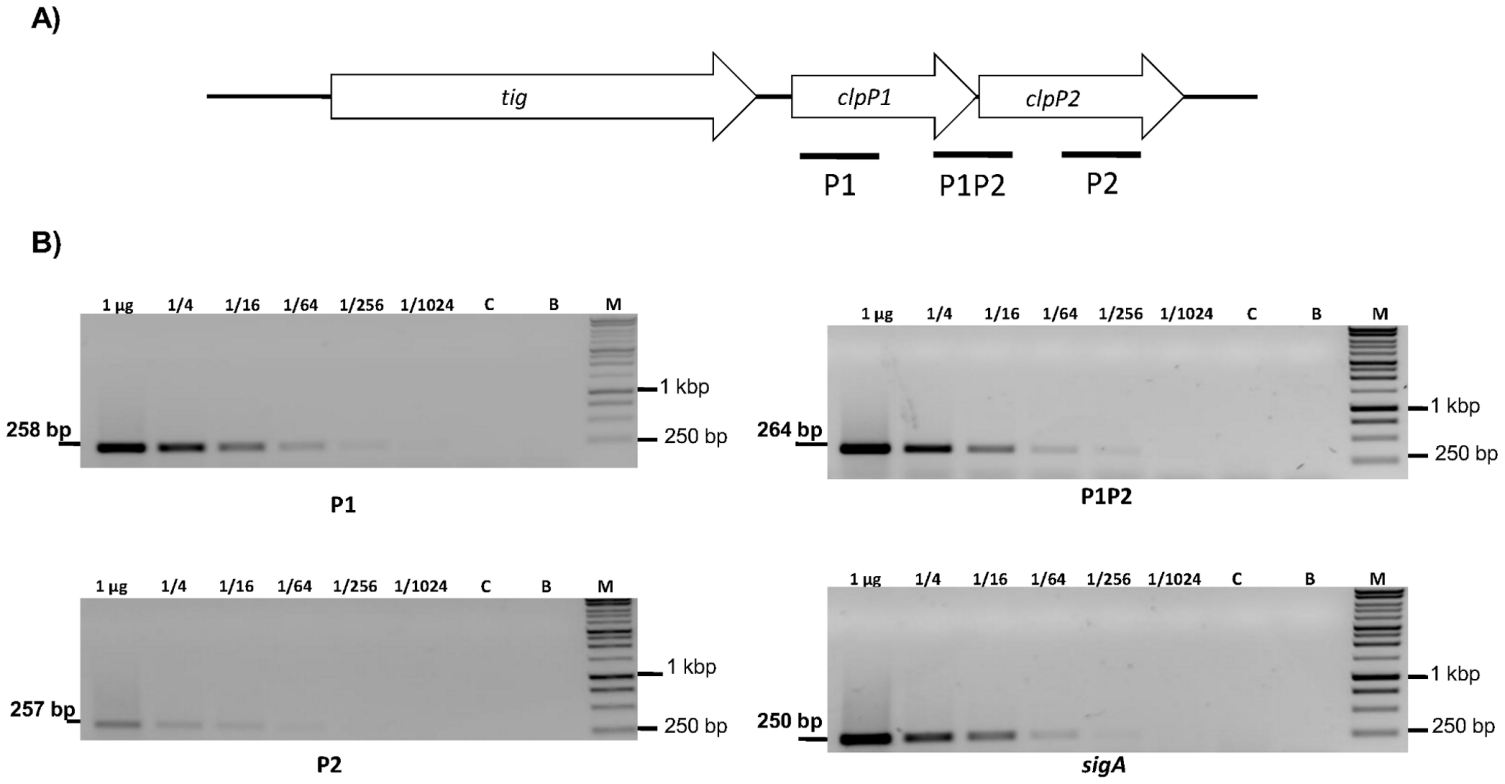
*clpP1* and *clpP2* are co-transcribed as an operon. A) Chromosomal organisation of *clpP1* and *clpP2*. Regions amplified for RT-PCR are marked. B) Limiting dilution semi-quantitiative RT-PCR. RNA was extracted from *M. tuberculosis* grown to late exponential phase in liquid cultures and cDNA was synthesised from 1 µg of RNA. Serial 4- fold dilutions of cDNA were used as a template for PCR using primers specific for *clpP1* (P1), *clpP2* (P2), the *clpP1*-*clpP2* junction (P1P2) and *sigA*. C: no RT control; B: no template blank, M: markers.

We wanted to determine if expression of the two ClpP subunits of *M. tuberculosis* could be independently regulated and thus be expressed under different conditions. From the genomic organisation it seemed likely that *clpP1* and *clpP2* would be co-expressed since they are organised in an apparent operon with overlapping stop and start codons. In order to determine if *clpP1* and *clpP2* are co-transcribed as an operon, we conducted semi quantitative RT-PCR (Fig. 1) to identify the mRNA transcripts. Primers were designed to amplify regions specific to *clpP1* (258 bp), *clpP2* (257 bp), or the *clpP1-clpP2* junction (264 bp). To determine the relative amounts of each mRNA species, limiting dilution RT-PCR was used as a semi-quantitative method; *sigA,* whose expression is considered to be constant across different conditions was used as a control [26]. Products were identified for all three mRNA species, indicating that the two genes are co-transcribed. The level of expression of the co-transcript and the *clpP1*-specific transcript were similar suggesting that *clpP2* was not being expressed from two promoters (since that would be expected to lead to higher levels of *clpP2* relative to *clpP1*). In addition, the relative levels of amplification of the *clpP2-*specific transcript derived from the 3′ end of the gene were lower compared to levels of *clpP1* mRNA (and as compared to the levels of *clpP1P2* transcript from the 5′ end of *clpP2*) as expected if the gene was at the 3′ end of an operon. These data strongly suggest that *clpP1* and *clpP2* are co-transcribed, in these conditions at least, under the control of a single promoter.

### Identification of the *clpP1*/*clpP2* Promoter

**Figure 2:**
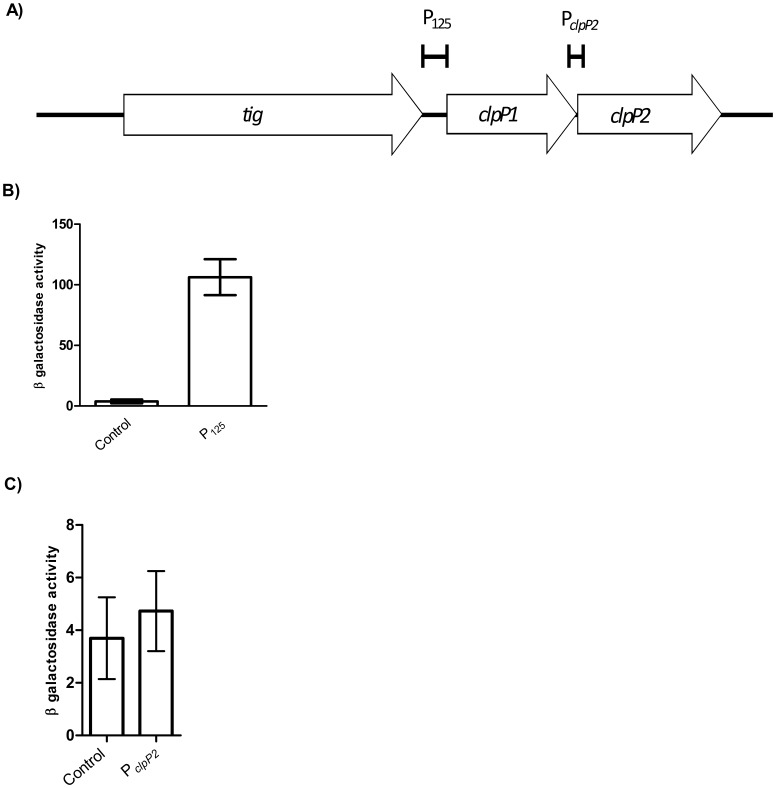
Identification of the promoter of the *clpP1P2* operon. A) The regions upstream of *clpP1* or *clpP2* tested for promoter activity are marked. B) P_125_ activity in *M. tuberculosis*. C) P*_clpP2_* activity in *M. tuberculosis*. Promoter activity was measured in transformants grown to late exponential phase in standing liquid cultures. Results are the average activity ± standard deviation of three independent transformants assayed in duplicate. Activity is given in Miller Units (MU)- measured as nmol of O-nitrophenol produced per min per mg of protein. Control = pSM128 empty vector control.

Since we had confirmed co-expression of the two genes, we wanted to identify the promoter of the operon. We cloned a 125 bp region (P_125_) encompassing the first two codons of *M. tuberculosis clpP1*, the intergenic region between *clpP1* and the upstream gene *tig,* and the *tig* stop codon (Fig. 2A), into pSM128 [21], an integrating vector present in a single copy in the genome; and introduced this in *M. tuberculosis*. pSM128 is a reporter plasmid containing a promoterless *lacZ* commonly used to measure gene expression in mycobacteria [27]–[29]. β-galactosidase activity was detected in aerobic cultures (Fig. 2B) (221 MU) confirming that a functional promoter was present in this region. To determine if there was a second promoter for *clpP2,* the region encompassing the first two codons of *clpP2* and 280 bp upstream of *clpP2* was also tested, but no activity was detected (Fig. 2C). This confirms that, at least under aerobic culture conditions, *clpP2* is not independently expressed from another promoter, so that *clpP1* and *clpP2* are co-expressed from the same promoter upstream of *clpP1*.

**Figure 3:**
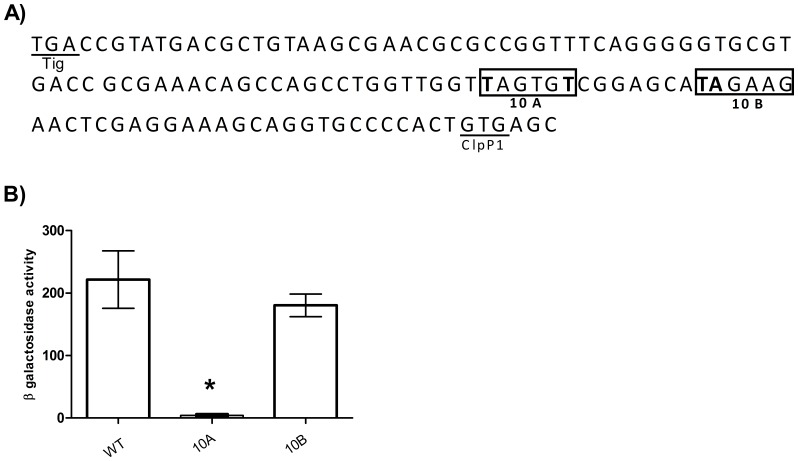
Identification of the −10 promoter element. A) Sequence of the P_125_ region upstream of *clpP1P2.* Putative −10 elements (10A and 10B) are boxed. The residues mutated are in bold. The predicted ClpP1 start codon and Tig stop codons are indicated. B) Identification of the −10 element. The following mutations were made - 10A: TAGTGT mutated to **C**AGTG**G**; 10B: TAGAAG mutated to **CG**GAAG. Results are the average activity of three independent transformants assayed in duplicate ± standard deviation. Activity is given in Miller Units (MU)- measured as nmol of O-nitrophenol produced per min per mg of protein. The background activity from pSM128 (control vector) was 4±2 MU. A significant difference, measured by the student’s t-test (unpaired, two sided), compared to the control vector (pSM128) is marked by an *(p<0.05).

Mycobacterial promoters are very diverse although the −10 element is frequently similar to the *E. coli* consensus sequence (TATAAT). We identified two putative −10 elements in the 125 bp promoter region (Fig. 3A). To determine if either of these are the genuine −10 element of the promoter, the TAGTGT hexamer (10A) was mutated to **C**AGTG**G** and the TAGAAG hexamer (10B) was mutated to **CG**GAAG and promoter activity was measured in *M. tuberculosis* (Fig. 3B). No significant difference in activity was observed when the TAGAAG hexamer was mutated, but when the TAGTGT was mutated to **C**AGTG**G,** promoter activity was abolished, indicating that the TAGTGT hexamer is the −10 element of the *clpP1P2* operon promoter.

### Promoter Activity was not Induced after Heat Shock or Oxidative Stress

Under stress conditions such as high temperature or oxidation, mis-folded proteins may be generated, which can further accumulate as protein aggregates and are potentially toxic for the cells. Clp proteases are involved in the degradation of mis-folded or aggregated proteins [30], [31] and are heat induced in several organisms including *Bacillus subtilis* and *C. glutamicum* [16], [32]. Additionally, oxidative stress, generated by addition of diamide, may induce *clpP1P2* expression in *M. tuberculosis* [10]. We tested the effect of similar stress treatments on P_125_ promoter activity in *M. tuberculosis*. We saw no difference in P_125_ promoter activity after oxidative shock or heat shock (data not shown) indicating that the promoter was not induced by these conditions.

### Identification of a Positive Regulatory Site in the Upstream Region

**Figure 4:**
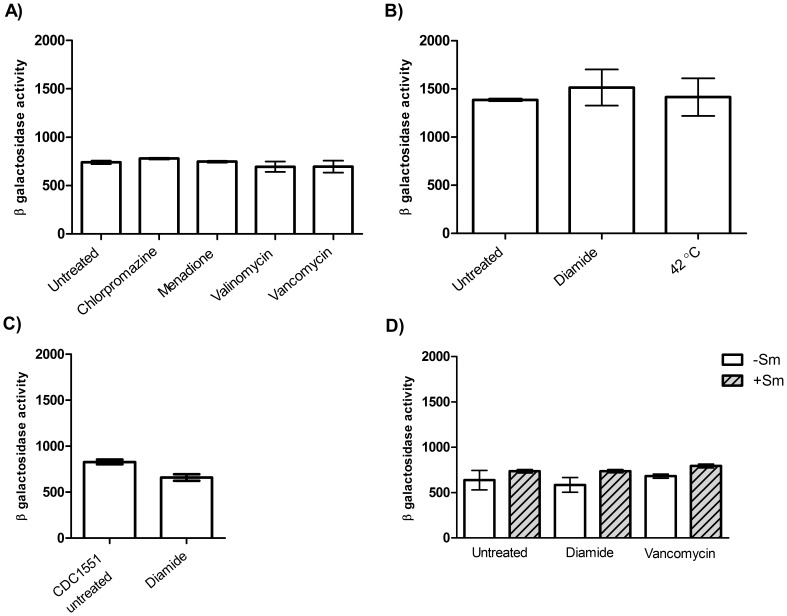
P_278_ promoter activity was not induced by stress treatments. A) P_278_ promoter activity in standing liquid cultures. Treatments were: 50 µg/mL of chlorpromazine for 3 h, 10 µg/mL of menadione for 3 h, 10 µg/mL of valinomycin for 3h, 6 µg/mL of vancomycin for 90 min. B) Promoter activity in rolling cultures. Treatments were: 42°C for 1 h, 10 mM diamide for 1 h C) Promoter activity in response to diamide treatment in *M. tuberculosis* CDC1551. D) Promoter activity in response to diamide and vancomycin treatments in presence or absence of streptomycin selection. Stress treatments were 10 mM diamide for 1 h or 6 µg/mL of vancomycin for 90 min. The average and standard deviation of three independent transformants assayed in duplicate is given. ß-galactosidase activity is given in Miller Units - measured as nmol of O-nitrophenol produced over time (min) per mg of protein. Activity is given in Miller Units (MU)- measured as nmol of O-nitrophenol produced per min per mg of protein. The background activity from pSM128 (control vector) was 6±3 MU under the different conditions tested.

Previous work had suggested that the *clpP* operon might be induced in response to oxidative stress [10], although we saw no evidence of upregulation of the promoter. We considered that the intergenic region between *clpP1* and *tig* might not contain all of the regulatory sites required for induction. To test this, we cloned a larger upstream fragment (278 bp - denoted P_278_) into pSM128 and measured promoter activity as before (Fig. 4A). P_278_ had promoter activity 6-fold higher than the shorter sequence suggesting that at least one binding site for a regulatory element was missing in the shorter region. We therefore used this construct for all other work.

Treatment with chlorpromazine, menadione, valinomycin in *M. tuberculosis* [33] and vancomycin in *M. smegmatis* [11] have previously been shown to increase ClpP mRNA levels. We tested each of these potential inducers in *M. tuberculosis*. P_278_ promoter activity was not induced or repressed in response to any of these treatments (Fig. 4A).

We also looked at P_278_ promoter activity in aerobically grown roller cultures of *M. tuberculosis* (Fig. 4B); once again no induction of activity was seen. However, we did note that promoter activity was higher in the highly aerated rolling cultures (1,380 MU) compared to standing cultures (740 MU).

We considered the possibility that induction of *clpP1P2* is strain-specific since Mehra and Kausal (2009) used *M. tuberculosis* CDC1551. P_278_ promoter activity was compared between *M. tuberculosis* H37Rv and *M. tuberculosis* CDC1551 but the activity of the CDC1551 strain (800 MU) was comparable to that observed in H37Rv and was not induced after diamide treatment (Fig. 4C) demonstrating that promoter induction was not strain specific.

Transcriptome profiling suggested that streptomycin exposure might induce *clpP1* and *clpP2* expression [33]. Since streptomycin was the antibiotic used to maintain selection pressure for the reporter plasmids, there was the possibility that the lack of induction may result from the fact that the promoter was already induced. Therefore, we measured P_278_ promoter activity without streptomycin selection and with diamide or vancomycin treatments; there was no significant difference in activity confirming that streptomycin was not pre-inducing *clpP1P2* promoter activity (Fig. 4D).

**Figure 5:**
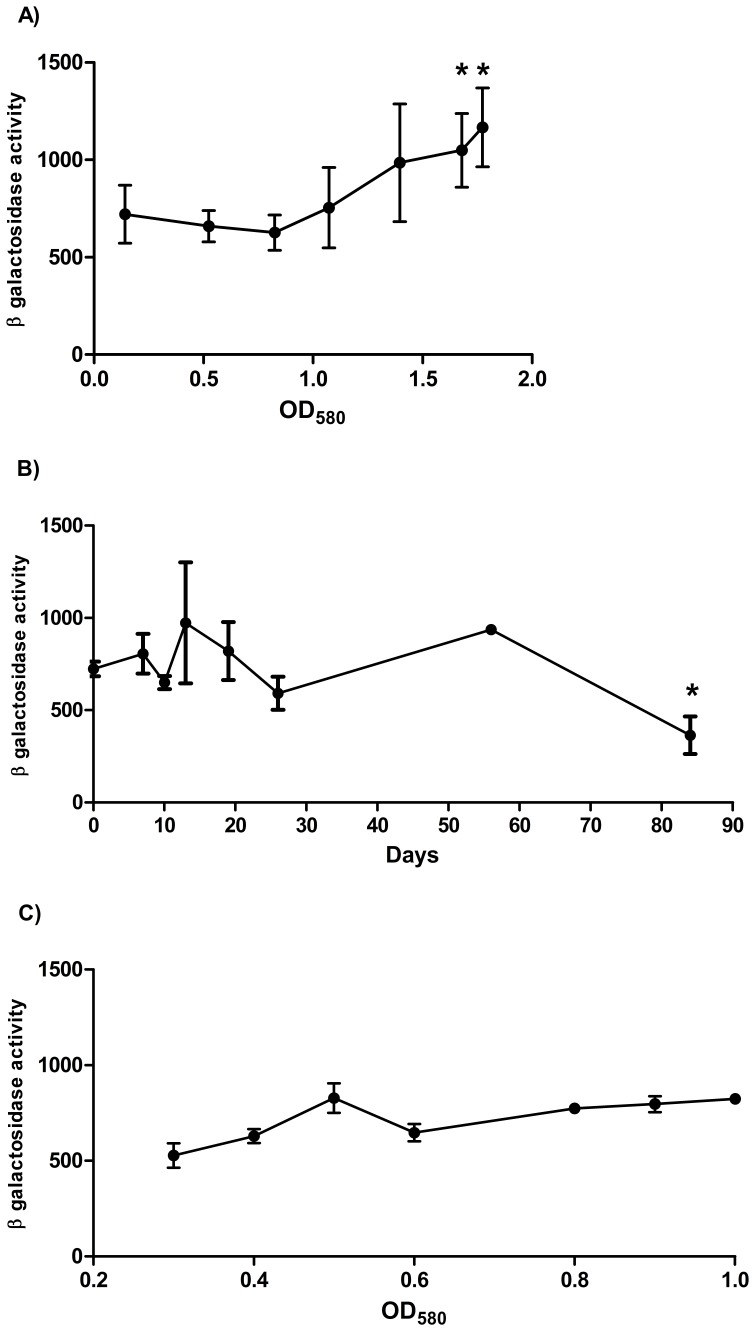
Promoter activity during aerobic growth, hypoxia, and reaeration in *M. tuberculosis.* A) *M. tuberculosis* transformants harbouring P_278_ were grown in aerobic culture. Results are the average activity of three transformants against average OD_580_. A significant difference, measured by the student’s t-test (unpaired, two sided), compared to promoter activity at OD_580_ = 0.15 is marked by an *(p<0.05). B) P_278_ promoter activity in the Wayne model of hypoxia. *M. tuberculosis* liquid cultures were inoculated to a theoretical starting OD_580_ of 0.004 in DTA medium. A significant difference compared to activity at day 0 is marked by an *(p<0.05) using the student’s t-test (unpaired, two sided). C) P_278_ promoter activity after reaeration. Long term hypoxic cultures (12 weeks) were used to inoculate medium and grown in aerobic rolling cultures. Cell-free extracts were prepared once the cultures reached an OD_580_ of 0.3. Results are the average activity of three independent transformants assayed in duplicate ± standard deviation. Activity is given in Miller Units- measured as nmol of O-nitrophenol produced per min per mg of protein.

The difference in promoter activity between rolling and standing cultures could indicate that the promoter activity is growth dependent. P_278_ promoter activity was measured from rolling aerated cultures over a time course from OD_580_ 0.15 to 1.7. The activity was constant from early log phase (OD_580_ = 0.15) to stationary phase (OD_580_ = 1.5) from 720 to 980 MU (Fig. 5A). However in late stationary phase (OD_580_ = 1.7 to 1.8), promoter activity increased significantly (to 1,050 MU) compared to the activity measured at early log phase (OD_580_ = 0.15) (Fig. 5A).

### Promoter Activity during Hypoxia and Reaeration

*M. tuberculosis* has the ability to survive inside the host environment for decades in a latent state before reactivation. The Wayne model is frequently used to study hypoxia [34], one condition believed to be encountered by the bacteria during latent infection, and inoculating hypoxic cultures into aerated medium (reaeration) can be used to mimic reactivation of the disease [17]. P_278_ promoter activity was measured during adaptation to hypoxia and over 12 weeks of survival in hypoxia (Fig. 5B). Promoter expression was similar between aerated and hypoxic cultures (typically around 700–800 MU) for up to 56 days (eight weeks) of hypoxia. There was no change in expression during the adaptation phase where cells switch from replicating into a non-replicating state. Promoter activity remained high during this state for several weeks, but was reduced approximately two-fold during long term hypoxia (8–12 weeks). After 12 weeks, hypoxic cultures were inoculated into aerated medium, and promoter activities measured once the cultures reached OD_580_>0.3 (it was not possible to measure activity in earlier cultures due to low cell numbers); promoter activity quickly returned to the higher level after reaeration (Fig. 5C). These data confirm that there is a small change in expression of the operon in response to long term hypoxia, although the relative levels of expression remained high.

### Mapping of a Positive Regulator Binding Site

**Figure 6:**
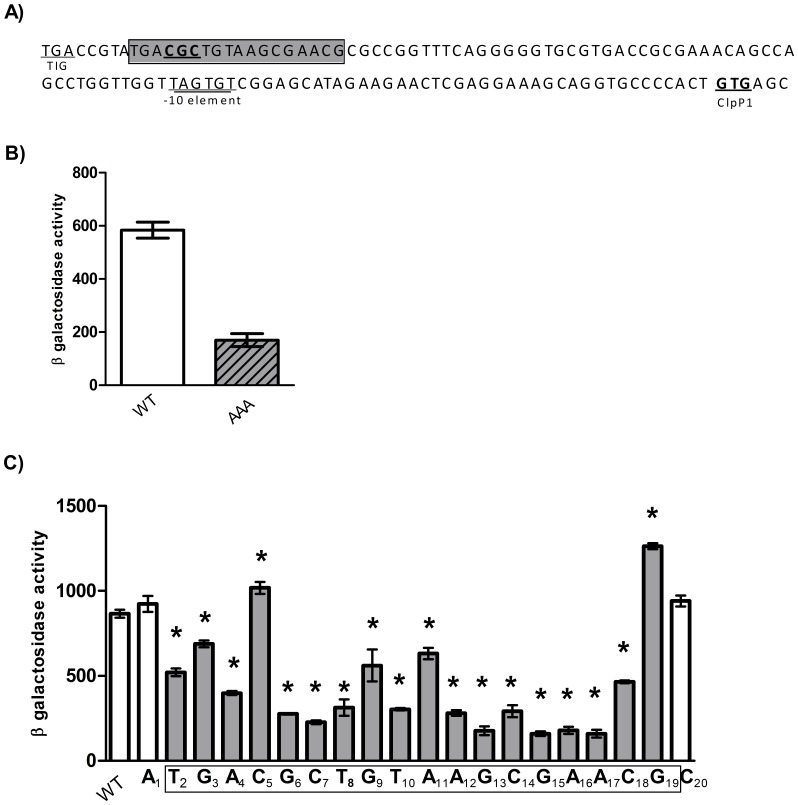
Identification of key residues in a regulatory region of the *clpP1P2* operon. A) Sequence of the region upstream of *clpP1P2*. The *tig* stop codon, −10 promoter element, and *clpP1* start codons are underlined. The 18 nucleotides that constitute the regulatory region are boxed in grey. The CGC region mutated is underlined in bold. B) Identification of a regulatory region. The CGC motif (underlined bold) was mutated to AAA. C) Mapping of a regulatory binding site. Single nucleotide substitutions in P_278_ were made by SDM. Residues A or T were mutated to G or and residues C or G were mutated to A. Results are the average activity of three independent transformants assayed in duplicate ± standard deviation. Activity is given in Miller Units- measured as nmol of O-nitrophenol produced per min per mg of protein. A significant difference of activity compared to wild-type P_278_ is marked by an *(p<0.05) using the student’s t-test (unpaired, two sided).

We found higher promoter activity using P_278_ as compared to P_125_ suggesting that the longer fragment contained at least one regulatory element binding site missing in the shorter fragment. Regulation of *clpP1P2* by the transcriptional activator ClgR is highly conserved within the actinomycetes [15], [16] including *M. tuberculosis* [17]. In all bacteria containing ClgR the central consensus motif CGC-N5-GCG is found upstream of *clpP1P2* and *clpC* [35]. This motif is found in the region upstream of *clpP1P2* in *M. tuberculosis,* approximately 100 bp upstream from the ClpP1 start codon (Fig. 6A). In order to determine if this was a true ClgR motif, we mutated the first half of the motif CGC to AAA in the P_278_ fusion. Promoter activity was significantly reduced after mutation (3.4-fold reduction) suggesting the possible binding of a regulator at this site, most likely to be ClgR (Fig. 6B).

We examined the extent of the proposed regulatory region further. Single nucleotide substitutions were constructed where A/T was mutated to G and C/G was replaced by A (Fig. 6C). Twenty bases were mutated in total and numbered 1 to 20. Two bases (A_1_ and C_20_) whose mutation had no effect on promoter activity were considered to be outside the regulatory region. Mutations in all of the other 18 bp (2–19) had a significant effect on promoter activity and we therefore considered these nucleotides to constitute the binding site, which is thus 18 bp long. Two mutations resulted in a higher promoter activity (C_5_A and G_19_A) while all other mutations resulted in a significant reduction in promoter activity.

### ClpP1 or ClpP2 Over-expression has Different Consequences

We hypothesised that the ClpP subunits might recognise different subsets of substrate proteins and so might play different roles in the cell. It was recently shown that the ClpP1P2 protease complex is required for the degradation of SsrA-tagged proteins in *M. tuberculosis*, which are abnormal proteins arising in presence of defective mRNAs [6].

The tmRNA system adds small peptide tags to proteins destined for degradation by the Clp protease. We wanted to determine which ClpP (or both) was responsible for the degradation of SsrA-tagged proteins. We generated LacZ variants where SsrA tags were fused at the C-terminal end; AANDENYA-LAA and AANDENYA-ASV protein tags were added to LacZ (referred as LacZ-LAA and LacZ-ASV respectively) and expressed under the control of the inducible acetamidase promoter (P_ami_) from *M. smegmatis* [23]. These degradation tags were previously shown to cause different protein half-lifes [36]. LacZ activity was measured in the wild-type background and in cells over-expressing *clpP1* and *clpP2* individually or together [5].

*M. tuberculosis* was grown under LacZ-induced (acetamide) or LacZ-uninduced conditions and steady state levels of LacZ protein were measured. The steady state level reflects a balance between protein synthesis and protein degradation, consequently if degradation of LacZ is performed by the ClpP proteases, a strain over-expressing the protease would have a higher protein turnover rate and lower steady state levels.

**Figure 7:**
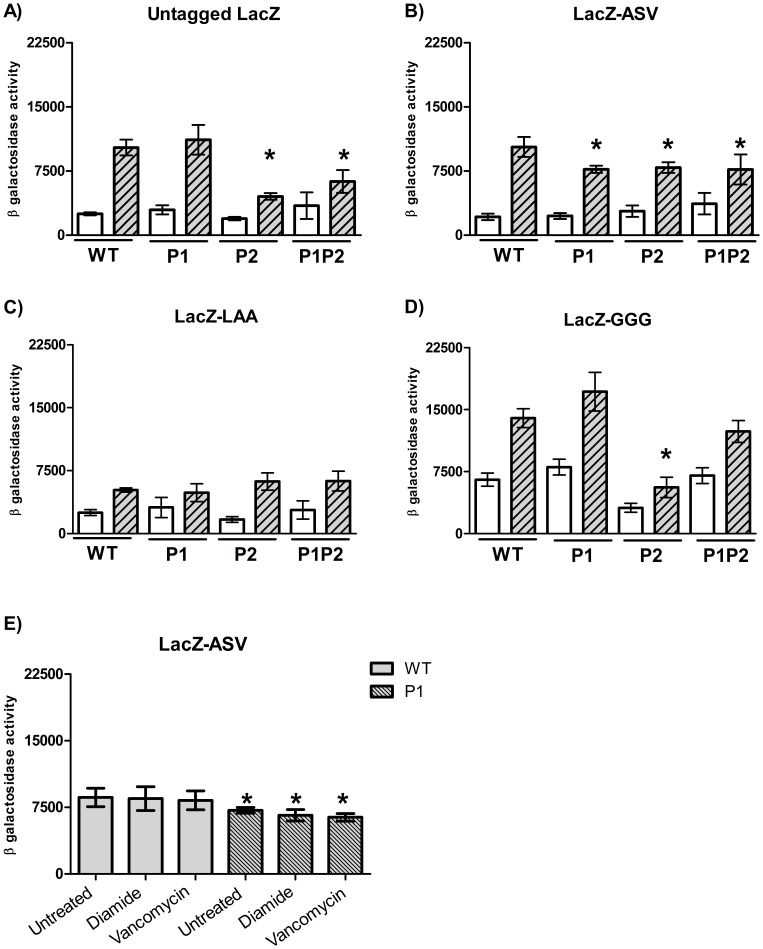
Protein turnover in strains over-expressing ClpP subunits. A to D) *M. tuberculosis* transformants were grown to late exponential phase in standing liquid cultures in presence of succinate +/− acetamide (0.1% w/v) and cell-free extracts were prepared and β-galactosidase activity measured. Empty bars: uninduced conditions (succinate); Grey striped bars: induced conditions (succinate+acetamide). A significant difference measured by the student’s t-test (unpaired, two sided) compared to the induced LacZ level in the WT strain is marked by an *(p<0.05). E) Three *M. tuberculosis* transformants carrying LacZ-ASV were grown to late exponential phase in standing liquid cultures in presence of acetamide (0.1% w/v) and cell-free extracts were prepared. Treatments were 10 mM diamide for 1 h or 6 µg/mL of vancomycin for 90 min. A significant difference from untreated WT is marked by an *(p<0.05). Results are the average activity of three independent transformants assayed in duplicate ± standard deviation. Activity is given in Miller Units- measured as nmol of O-nitrophenol produced per min per mg of protein. Strains are- WT: wild-type; P1: over-expressing ClpP1; P2: over-expressing ClpP2; P1P2: over-expressing ClpP1 and ClpP2.

We confirmed that the expression of LacZ was acetamide-inducible: untagged LacZ activity was 4.1-fold higher under induced conditions in the wild-type strain (Fig. 7A); the tagged versions were also induced (2–4 fold), although induction of LacZ-LAA was only 2-fold. The level of LacZ-ASV was comparable to untagged LacZ, with similar induction levels, confirming that the tag alone did not change turnover under normal levels of ClpP expression (Fig. 7B). Interestingly, the addition of the LAA tag resulted in reduced steady state levels of LacZ even in the wild-type background, suggesting that this tag is recognised by the native ClpP protease and that degradation of LacZ-LAA is occurring (Fig. 7C). Replacing the three terminal residues of the tag, which are critical for recognition, resulted in loss of degradation – the levels of LacZ were comparable to the untagged protein (Fig. 7D).

### ClpP2 Over-expression Induces Degradation of Untagged LacZ

Clp proteases are responsible for degrading proteins tagged by the tmRNA system, but can also degrade untagged proteins. We looked at the effect of over-expressing either ClpP1 or Clp2, or both. No difference in the steady state levels of untagged LacZ activity was observed when ClpP1 was over-expressed (Fig. 7A). However, there was a significant decrease in the steady state levels of untagged LacZ when ClpP2 or ClpP1-ClpP2 was over-expressed (2.3-fold and 1.6-fold reduction respectively). These data suggest that ClpP2 is involved in degrading untagged proteins.

### ClpP1 and ClpP2 Over-expression Induce Degradation of ASV Tagged LacZ

Incorporation of the ASV tag changed the dynamics of protein turnover. There was a significant decrease in steady state levels of LacZ-ASV when ClpP1 or ClpP2 were over-expressed independently or together (1.3-fold reduction in all over-expressing strains) (Fig. 7B). This suggests that the SsrA tag directs proteins to degradation by both ClpP1 and ClpP2 in *M. tuberculosis*.

### LAA Tagged LacZ is Degraded Quickly

The terminal three residues of the *M. tuberculosis ssrA*tag sequence are LAA [37]. Tagging LacZ with LAA had a profound effect on protein activity levels. A reduction in steady state quantities of LacZ-LAA was seen in all strains, even in the WT as compared to untagged LacZ (Fig. 7C); LacZ-LAA activity was approximately 5,000 MU in induced conditions in all strains, while untagged and LacZ-ASV activity was about 10,000 MU in the WT strain. Since LacZ-LAA is degraded more quickly than LacZ or LacZ-ASV, this suggests that the LAA tag is more efficient at directing proteins into the degradation pathway. Over-expression of ClpP1 and ClpP2 had no effect on steady state levels of LacZ-LAA, presumably because the turnovers rates were already at their maximum.

### ClpP1 Recognises the Last Three Residues of the Tag

The *E. coli* ClpXP complex recognises the last three amino acids of the *ssrA* tag (AANDENYA**LAA**). To determine if the last three amino acids of the tag were also the determinant for recognition in mycobacteria, the last three residues were mutated to GGG. Interestingly, the uninduced level of LacZ-GGG (7,400 MU) was higher compared to either untagged or tagged LacZ (Fig. 7D). The steady state levels of LacZ-GGG were similar between the wild-type and ClpP1 over-expressing strains confirming that the protein was not being recognised by ClpP1 for degradation and that the *ssrA* tag is important for ClpP1-mediated degradation. In contrast, LacZ-GGG steady state levels were significantly reduced when ClpP2 was over-expressed (2.5-fold reduction) confirming that ClpP2 degrades abundant proteins in the absence of a C-terminal degradation signal, as also seen with the untagged protein. Interestingly in this case, when both *clpP1* and *clpP2* were over-expressed the steady state levels were unchanged.

### Proteolytic Turnover is not Increased after Heat or Oxidative Shocks

Since LacZ-ASV appeared to be a good indicator of ClpP1 and ClpP2 activity, LacZ-ASV steady state levels were measured after oxidative shock (addition of diamide), or vancomycin treatment in order to determine if these conditions increased protein turnover (Fig. 7E). *M. tuberculosis* transformants carrying LacZ-ASV were grown in the presence of acetamide to induce expression and steady state levels of LacZ were measured after stress treatments. There was no significant difference in LacZ-ASV steady state levels between untreated and treated cultures suggesting that protein turnover is not increased in these conditions. As seen before LacZ-ASV levels were reduced when ClpP1 was over-expressed. However no increase in LacZ-ASV degradation was observed after stress treatments, suggesting that ClpP1 activity is not increased at the functional level.

## Discussion

We were interested in determining the functional significance of the presence of two ClpP proteases in *M. tuberculosis* and whether they play different or overlapping roles in proteolytic degradation. *ClpP1* and *clpP2* are co-transcribed under the control of a single promoter, excluding the possibility they are expressed under different conditions.

*M. tuberculosis* encodes 13 different putative sigma factors which ensure promoter recognition [38]. The −10 element of the *clpP1P2* promoter was identified and this sequence matches the consensus sequence for the binding sequence of sigma factor A, the principal sigma factor of *M. tuberculosis* [39]. The −10 element sequence of the *clpP1P2* operon (TAGTGT) has three nucleotides in common with the *E. coli* consensus sequence (TATAAT), and as expected the intrinsic activity of the promoter element deprived of regulator binding sites (92 bp) was very low (12 MU; data not shown).

We tested the promoter constructs in related mycobacterial species. Interestingly promoter activities were significantly higher in *M. tuberculosis* compared to the model organisms *M. smegmatis* or *M. marinum* (data not shown) often used as genetic hosts for *M. tuberculosis* studies. A possible explanation for this may be the absence of regulatory control elements in the model organisms. We found that the high level expression of *clpP1P2* required the presence of a regulatory motif consistent with ClgR regulation which might operate differently in the fast growing species.

We mapped the extent of a regulatory region for activity; the 18 bp sequence identified is an imperfect palindrome which matches the consensus sequence of the ClgR binding site in *C. glutamicum* [40]. ClgR binding upstream of *clpP1P2* has been demonstrated in *M. tuberculosis* [17], although the binding motif was not characterised, strongly suggesting that this region is the sequence where ClgR is binding. Although the regulatory region is present in P_125_ and P_278_, the two regions had different activity, with much higher promoter activity in the larger fragment. This could be due to presence of a terminator in the pSM128 plasmid close to the regulatory site which alters the binding of the regulator in the shorter construct.

Promoter activity was slightly increased in late stationary phase (after an OD_580_ of 1.7) suggesting that an increase in the Clp protease activity may be necessary to degrade proteins no longer required for growth. The importance of the Clp proteases in stationary phase was previously demonstrated as *E. coli* and *B. subtilis clpP* mutants have a loss of viability during stationary phase survival [32], [41].

Promoter activity during hypoxia was high and constant for up to eight weeks. This is an unusual pattern of activity, since the expression of most genes is typically down-regulated or turned off completely during hypoxia when metabolic activity is greatly reduced. These data are consistent with our previous report of sustained high level expression of *clpP1* and *clpP2* at the mRNA level in a hypoxic environment [9]. It is likely that there is a high level of protein turnover during adaptation to hypoxia and the controlled shift down to a non-replicating state and the high level of expression of ClpP1P2 suggests an important role for the Clp protease under these conditions.

Promoter activity was reduced between 8 to 12 weeks of hypoxia but returned to its original activity quickly after reaeration confirming previous reports of *clpP* induction during reaeration [17]. Reaeration has been used as a model of reactivation of latent infection; the rapid induction of promoter activity during reaeration may suggest a role for the Clp proteases during reactivation. Targeting Clp activity, either directly or via ClgR, could therefore be a novel and attractive approach to prevent *M. tuberculosis* survival during infection and avoid reactivation of the disease.

The role of proteases is particularly vital during stresses that increase the occurrence of damaged proteins. *clpP* induction under heat shock conditions is variable among actinomycetes: while *clpP1P2* expression is induced upon severe heat stress in *C. glutamicum* [16], none of the *clp* genes are heat induced in *Steptomyces lividans* [15]. We did not detect any increase in promoter activity in *M. tuberculosis* during heat shock at 42°C; this is consistent with previous microarray studies which did not detect any induction of *clpP1* or *clpP2* following heat shock [42].

We saw no induction of promoter activity after diamide or vancomycin treatments [10], [11]. Previous work had suggested that diamide may induce *clpP1P2* expression and that this was regulated by SigH. However this study compared the expression levels of *clpP1P2* in a wild-type strain to expression levels in a *sigH* deletion strain after diamide treatment (and expressed them as a ratio). No measurement of mRNA was made in the wild-type strain; the apparent over-expression in the wild-type strain in the presence of diamide could instead result from under-expression in the *sigH* deletion strain (since data were expressed as a ratio). The induction by vancomycin was seen in *M. smegmatis*; we often see differences in gene regulation between this non-pathogenic, fast-growing strain and the pathogenic, slow-growing *M. tuberculosis* and in fact the promoter identified in this study had different activity between the two species, so there may be true differences in regulation.

ClpP1 and ClpP2 play a role in protein quality control by degrading SsrA-tagged proteins. In order to determine if a difference in substrate recognition was seen between the two ClpP proteins, unstable LacZ variants with different degradation tags were used. The first eight amino acids of the tags were similar (AANDENYA) and the last three residues differed (LAA, ASV, GGG). We used the acetamidase promoter to generate high level expression of the reporter LacZ, since over-expression of proteins often makes them vulnerable to proteolytic degradation as the cells attempt to minimise metabolic burden. No changes in LacZ levels were observed in the non-induced conditions suggesting that degradation only occurs when protein concentration reaches a threshold level. When ClpP1 and ClpP2 were over-expressed individually or together, LacZ-ASV steady levels were significantly reduced, demonstrating that both ClpP1 and ClpP2 are involved in the degradation of proteins harbouring this C-terminus tag. However, degradation was not increased during heat or oxidative stresses.

Mutation of the last three residues of the SsrA tag prevented recognition by ClpP1, demonstrating that these residues were the determinant for identification. These residues are recognised by the ClpXP complex in *E. coli* suggesting that ClpP1 and ClpP2 can interact with ClpX for the degradation of SsrA-tagged proteins in *M. tuberculosis*. The adaptor protein SspB recognises the AANDENY portion of the tag which is different from the *M. tuberculosis* sequence [44], [45]. *M. tuberculosis* does not have a SspB homolog [46] and ClpP1 and ClpP2 proteases recognise *E. coli* tag sequences suggesting that an accessory protein may not be necessary for degradation in *M. tuberculosis* as it is the case in *S. pneumoniae* [47].

Interestingly, over-expression of ClpP2 led to increased turnover of untagged LacZ demonstrating that ClpP2 can degrade proteins independently of the tag sequence. Over-expression of ClpP1 did not lead to increased turnover of untagged LacZ. This difference in substrate degradation suggests that ClpP1 may be more specific to a particular set of substrates, such as proteins harbouring the SsrA degradation tag, as opposed to ClpP2 that might be responsible for a general and central housekeeping function. To function as an effective protease ClpP1 and ClpP2 associate; however the levels of ClpP1 appear higher than of ClpP2 (Fig. 1), therefore under ClpP2 over-production conditions the observed activity of ClpP2 may be seen due to its previous association with ClpP1.

Clp ATPases are the subunits involved in substrate recognition implying that the difference in substrate specificities displayed by the two ClpP proteases is likely to result from ClpP1 and ClpP2 interacting with different ATPase subunit(s). As both ClpX and ClpC1 are present in *M. tuberculosis* an attractive model would be that ClpX could interact with both ClpP1 and ClpP2 so they can degrade SsrA-tagged proteins, while ClpC1 could only interact with ClpP2 for the degradation of a specific set of substrates. *M. tuberculosis* ClpP2 interaction with ClpC1 has been previously demonstrated, and RseA was shown to be targeted by the ClpC1P2 complex [11], [48], both confirming the formation of this complex in the cell and also its ability to degrade specific untagged proteins. We attempted to demonstrate protein-protein interactions using two alternative bacterial two hybrid systems for all combinations of ClpP and ATPase subunits (and accessory proteins), but neither system allowed us to identify any interactions. This is likely due to a limitation of such systems in detecting interactions which form complexes, since we were also unable to detect interactions between the *E. coli* subunits.

Recent work suggests that the functional proteolytic unit of the Clp protease is comprised of both ClpP1 and ClpP2, with seven subunits of each forming one of the two heptameric rings and that neither ClpP1 nor ClpP2 are active when expressed independently [6]–[7]. This contrasts with earlier work demonstrating that ClpP2 alone could form a functional protease with ClpC1 and effect degradation of RseA [11]. Since both studies were conducted with recombinant (tagged) proteins *in vitro* or using a surrogate host (*M. smegmatis*), they may not truly reflect the intracellular setting in *M. tuberculosis*. However, both studies demonstrate that the ClpP1 and ClpP2 have different specificities, which is in agreement with our findings.

The two ClpP subunits are co-expressed and are therefore present in the cell under the same conditions. The specificity of the two subunits seem however to be different; we propose that ClpP1 is involved in the degradation of SsrA-tagged proteins only, while ClpP2 has a more general role in proteolysis being able to degrade SsrA-tagged or untagged protein substrates.
